# Fracture strength evaluation for different posts in endodontically treated teeth

**DOI:** 10.6026/973206300223476

**Published:** 2026-06-30

**Authors:** Manmeet Kaur, Shanta Chopra, Unnati Nautiyal, Alka Gagneja, Jasmeet Kaur, Mohini Garg

**Affiliations:** 1 Department of Prosthodontics Crown and Bridge, LuxmiBai Institute of Dental Sciences and Hospital,Patiala, Punjab, India; 2 Department of Prosthodontics, Crown & Bridge and Oral Implantology, Genesis Institute of Dental Sciences and Research, Ferozpur, Punjab, India; 3 Department of Conservative Dentistry and Endodontics, Faculty of Dental Sciences, SGT University, Gurugram, Haryana, India; 4 Department of Conservative Dentistry and Endodontics, Shaheed Kartar Singh Sarabha Dental College and Hospital, Ludhiana, Punjab, India; 5 Department of Conservative Dentistry and Endodontics, Geethanjali Dental & Research Institute, Udaipur, Rajasthan, India; 6 Department of Prosthodontics, Maharishi Markandeshwar College of Dental Sciences and Research, Mullana, Ambala, Haryana, India

**Keywords:** Endodontic tooth, fracture resistance, post and core

## Abstract

Management of endodontically treated teeth with severe loss of coronal structure is challenging, fracture resistance of such teeth can be improved using various types of post and core restoration. Therefore, it is of interest to evaluate the fracture resistance of different post systems in endodontically treated. Extracted maxillary incisors after endodontic treatment were randomly divided into 4 groups based on type of post used. Compressive load required to fracture the post treated tooth was measured using a universal testing machine. Thus, zirconia posts showed greatest fracture resistance compared to other tested post systems.

## Background:

The main etiological factors that cause coronal tooth structural loss are caries and trauma [1]. The loss of tooth structure may affect mastication and esthetic. The remaining tooth structure is the most crucial factor for the good prognosis. The success of the endodontically treated teeth depends mainly on the post endodontic restoration than the endodontic treatment quality as suggested by Ray and Trope [2]. Endodontically treated teeth are traditionally restored with a post-core and crown system which depend mainly on the design, material and modulus of elasticity of dental posts. Post plays a major role in retention and fracture resistance of coronal restoration of endodontically treated teeth [3]. In many cases, posts are necessary to properly preserve the core material in teeth that have suffered severe hard tissue loss as a result of trauma or cavities [4]. Endodontic post can be divided into prefabricated and customer made metallic and non-metallic (tooth colored) post [5]. The use of prefabricated post systems allows for a wide range of post systems, from the traditional custom cast post and core to one-visit method [6]. The amount of residual dentin and tooth canal shape play critical roles in the strength and resistance of a tooth with posts. Hence, a post is not commonly used in teeth with flared canals [7]. The need for endodontic posts and core materials with exceptional esthetics has increased due to the expanding usage of all ceramic dental restorations. Dentin and enamel that are undamaged have elastic modulus of about 15 Gpa and 80 Gpa, respectively [8]. It has been suggested that the same elastic modulus (18 Gpa) of the root dentine and fiber post can reduce stress concentration [9]. Endodontically treated teeth (ETT) are restored by the conventional metal post, fiber post. In the past, they were restored by porcelain fused to metal Richmond crown. However, evidence has shown that fiber post, resin core and resin filling are better alternatives than the metal post [10]. Fiber posts contain either carbon fibers or quartz fibers, embedded in epoxy or methacrylate resin matrix. The fibers are parallel to the long axis of the post with diameter ranging between 6 to 15 mm2. While the number of fibers range between 25 and 35 per mm2 in respect to post type [11]. Custom-made tooth-colored posts include ceramic and zirconia posts. They are used when high esthetic demand is required in the anterior zone of teeth to prevent long term discoloration [11]. Zirconia post and core system offers chemical stability and its similarity to natural tooth structure. Polyether ether ketone (PEEK) is a thermoplastic material that represents a viable biomaterial. It is not only able to replace conventional polymers, but also metals, alloys and ceramics in the field of dentistry [12]. Polyether ether ketone offers a good combination of high tensile strength, stiffness, toughness, wear resistance, low coefficient of friction, excellent chemical resistance and very low moisture absorption with a high melting point. PEEK is shown effective core materials [13]. Therefore, it is of interest to describe the fracture resistance of different post systems in endodontically treated.

## Materials and Methods:

Total 40 maxillary central incisors with complete root and without any pathology extracted for periodontal purpose were included for study. Teeth were cleared from periodontal fibers, washed, sterilised and stored in saline until use. Root canal treatment and obturation was done for these teeth. Crown size was reduced to middle third to mimic lost crown structure. The specimens were mounted in acrylic resin blocks, with the long axis of the block, midfacial extent of each tooth parallel to the long axis of the block. The mid- facial extent of cementoenamel junction located 2 mm coronal to acrylic resin. Root length for posts was standardized by reducing the crown of each tooth to a height of 1 mm over the cementoenamel junction. Later these teeth were randomly divided into 4 groups based on type of post used. Group I-quartz fiber post, Group II, - zirconia posts; Group III- everStick posts and Group IV- glass fiber post. After post space preparation each posts were cemented using dual cure resin followed by core buildup with composite. Compressive load required to fracture the tooth was measured using a universal testing machine. The obtained data was statistically analysed using SPSS statistical software version 25.0 using Tukey's post-hoctest at P-value of less than 0.05.

## Results:

The compressive strength of zirconia posts was highest (698.35 ± 26.321) followed by quartz fiber post (656.38 ± 31.376), ever Stick posts (621.24 ± 25.354). Lowest compressive strength was exhibited by glass fiber post (562.46 ± 22.234). An analysis of variance revealed a statistically highly significant difference (P < 0.005) among the different posts used. There was statistically significant difference between carbon posts vs. zirconia posts, carbon posts vs. everStick posts and zirconia posts versus everStick posts (P < 0.05) (Table 1). Zirconia posts with highest fracture resistance indicate its application in stress bearing area. Posts with lowest fracture resistance are not suitable for higher stress bearing area rather they can be indicated for lower stress areas. Improved fracture strength is significant in longevity of the restored tooth.

## Discussion:

The rehabilitation of extensively damaged teeth with no dentinal support at the coronal portion of the root canal is very difficult [14]. The present *in vitro* study was attempted to compare the fracture resistance and the mode of failure of endodontically treated teeth restored with different post types. We found highest fracture resistance with zirconia post compared to other type. Maxillary premolars with non-carious cervical lesions that were endodontically repaired using PFCs crowns demonstrated greater fracture resistance than maxillary premolars without posts, according to Biswas *et al.*'s evaluation of the fracture resistance of endodontically treated teeth with non-carious cervical lesions (NCCLs) using various types of aesthetic posts [15]. According to Jakkamsetty *et al.* when compared to prefabricated glass fiber posts, the fracture resistance of endodontically treated teeth strengthened with hybrid posts shown substantial values [16]. Post endodontic restorations might fail for a variety of reasons. The use of large-diameter posts increases the risk of apical or lateral perforation and is the primary cause of complications [17]. Dghaily *et al.* used zirconia and polyether ether ketone (PEEK) materials to examine the fracture resistance of many treatment procedures meant for teeth that had been mutilated. They came to the conclusion that polyether ether ketone might offer endodontically treated teeth a dependable therapeutic alternative [1]. Saad *et al.* assessed the fracture resistance of teeth that had undergone endodontic treatment and were repaired using two post-core systems with varying post-space diameters. They came to the conclusion that post space diameter affected endodontically treated teeth's ability to withstand fractures. The fracture resistance was significantly impacted by the post material's modulus of elasticity [18]. Zirconia posts demonstrated the highest fracture resistance compared to carbon and EverStick posts, according to Moyin *et al.* [19]. According to Makade *et al.* teeth that were endodontically treated but lacked a post-core system had the lowest fracture resistance, indicating that the tooth needed to be reinforced [20]. Malekipour *et al.* examined two types of posts and composite resin for structural reinforcement in teeth with structural compromise. The non-compromised samples had the highest fracture resistance values, while the compromised samples had the lowest values that were only recovered by the resin composite [21]. According to a systematic review by Badami *et al.* prefabricated supports made of titanium and stainless steel placed more stress on the rebuilt tooth structure, with the cervical and apical thirds of the root experiencing the most stress. Additionally, prefabricated zirconia displayed greater stress on the restored tooth, with the middle third of the root exhibiting the highest concentration of stress [22]. Aly *et al.* concluded that, bundle fiber posts had comparable fracture resistance values in comparison to tapered fiber posts [23]. Kaur *et al.* stated that, short fiber-reinforced composite proved to have superior properties compared to bulk-fill composite resin, direct composite (PRC) resin and dual-cure composite (DCC) [24]. Ozyurek *et al.* found highest fracture resistance in the control group and the lowest with post core with CAD/CAM crowns [25]. The limitation of the study is smaller samples size and *in vitro* study; this study may not accurately reflect the situation *in vivo* as the fracture resistance was determined by applying heavy load to a single point. Further *in vivo* studies are needed to validate the result.

## Conclusion:

Fracture resistance of endodontically treated teeth can be improved with various posts systems. In the present study Zirconia posts showed the maximum fracture resistance compared to carbon posts, everStick posts and glass fiber posts. Hence Zirconia posts can be suggested for better clinical outcome.

## Advancement to knowledge:

The fracture strength of endodontically treated teeth (ETT) are; there shift toward optimizing the mono block effect where the post, cement and dentin function as a single unit, Glass Fiber Posts provides excellent esthetics and favorable, repairable fractures. Recent advancements highlight that zirconia posts offer superior fracture resistance.

## Figures and Tables

**Table 1:** Mean compressive strength exhibited by different posts before fracture.

**Post type**	**Mean ± SD**	**p**
Group I- quartz fiber post	656.38 ± 31.376	
Group II - zirconia posts	698.35 ± 26.321	
Group III- everStick posts	621.24 ± 25.354	
Group IV- glass fiber post	562.46 ± 22.234	0.001
